# Stability and Perpetuation of Human T‐Lymphotropic Virus 1 (HTLV‐1) in Intrafamilial Transmissions of Infection in an Endemic Region of the Brazilian Amazon: A Pilot Study

**DOI:** 10.1155/jotm/2728551

**Published:** 2025-12-29

**Authors:** Cássia Cristine Costa Pereira, Milena Cristina Martins da Silva, Samir Mansour Moraes Casseb, Maria de Nazaré Lima dos Reis, Louise de Souza Canto Covre, Edna Aoba Yassui Ishikawa, Leonardo Miranda dos Santos, Carlos Araújo da Costa, Maisa Silva de Sousa

**Affiliations:** ^1^ Cellular and Molecular Biology Laboratory, Center for Tropical Medicine, Federal University of Para, Belem, Para, Brazil, ufpa.br; ^2^ Center for Oncology Research, Federal University of Para, Belem, Para, Brazil, ufpa.br

**Keywords:** Amazon, HTLV-1, intrafamilial transmission, vertical transmission

## Abstract

**Introduction:**

Human T‐lymphotropic virus 1 (HTLV‐1) is related to several clinical diseases, including adult T‐cell leukemia/lymphoma and HTLV‐1‐associated myelopathy. Intrafamilial transmission of HTLV‐1 is rarely reported in Brazil and worldwide.

**Objective:**

To identify HTLV‐1 family transmissions in Belem, Para, the Amazon region of Brazil.

**Methods:**

Nested PCR of the pX gene, followed by restrict fragment length polymorphism (RFLP) to identify HTLV‐1. Nested PCR of the 5′LTR region was used for nucleotide sequencing. Nucleotide sequences were analyzed using GENEIOUS 4.8.5 and MAFFT V.7.

**Results:**

The study investigated 72 individuals (14 index cases and 58 relatives) from 14 families, identifying HTLV‐1 infection in 47% (27/58) of the relatives. Vertical transmission occurred in 14 (39%) of the 36 mother/child relationships investigated, and sexual transmission was observed in 14 (74%) of the 19 marital relationships (*p* = 0.0299). Both transmission routes were identified only in families with the highest average number of relatives investigated. Nucleotide analysis demonstrated HTLV‐1 stability in intrafamilial transmission.

**Conclusion:**

High rate of HTLV‐1 intrafamilial transmission was identified in this study. Sexual and vertical transmission are important to familiar dissemination and perpetuation of the virus in this endemic area of the Brazilian Amazon.

## 1. Introduction

Human T‐lymphotropic virus 1 (HTLV‐1) was identified in the early 1980s in patients with cutaneous lymphoma and adult T‐cell leukemia [1]. Although three other types of HTLV exist (Types 2–4), only HTLV‐2 is rarely associated with hairy cell leukemia or myelopathy [2]. Several late lymphoproliferative disorders may be associated with HTLV‐1 infection, such as adult T‐cell leukemia/lymphoma (ATL) [3] and tropical spastic paraparesis/HTLV‐1‐associated myelopathy (TSP/HAM) [4], in addition to cutaneous and ophthalmological manifestations and rare events of polymyositis, bronchoalveolar pneumonia or keratoconjunctivitis [5, 6]. Approximately 10% of people living with HTLV (PLHTLV) progress to TSP/HAM or ATL [7].

Transmission can occur through contaminated body fluids. In unprotected sexual contact, transmission from men to women is predominant due to the higher concentration of cells permissive for this virus in seminal fluids [8]. Furthermore, transmission is significant via the transplacental route [9] and through blood transfusion and blood products [10, 11]. Vertical and/or intrafamilial transmission of HTLV‐1 occurs mainly through breastfeeding [12, 13] in up to 25% of children of seropositive mothers [14, 15].

In Brazil, there are between 800,000 and 2.5 million people infected with HTLV‐1 [16, 17], which has been a neglected infection due to its silent epidemiology and little attention from public authorities [18, 19]. In the state of Para, in the Amazon region of Brazil, HTLV‐1 infection has varying frequencies among cities; residents from Ananindeua, Marituba, and Benevides in the metropolitan region of Belem (0.35%) [20]; riverside dwellers (1.14%) [21]; rural communities (0.7%) [22]; HIV co‐infected individuals (1.4%) [23]; blood donors (28.6%) [24]; and patients with HTLV referred to the health education unit for diagnosis and multidisciplinary monitoring of PLHTLV in the city of Belem (72.9%) [25], passers‐by in public places in Belem (1.4%) [26, 27], and in remnants of quilombos (0.9%) [28].

Vertical transmission of HTLV‐1 in family clusters has been documented in Brazilian populations [29, 30]. Annually, 16,548 HTLV‐1‐positive women become pregnant [30], with frequencies of vertical transmission ranging from 36.3% in Salvador [31], 22% in central Brazil [32], and 25.6% in Belem [25]. The long latency, in many cases with a subclinical profile or nonspecific symptoms of virus‐related diseases, associated with the high rate of familial aggregation of the infection, is a determinant for the silent dissemination of HTLV‐1 [5].

Studies focused on the characteristics of intrafamilial transmission with the support of viral genetic data are fundamental to understanding the epidemiology and direct diagnostic and prevention measures for HTLV‐1 infection and related diseases. Our study evaluated the intrafamilial transmission of HTLV‐1 in the state of Para, a region in the Brazilian Amazon endemic for this infection.

## 2. Materials and Methods

### 2.1. Study Population

This is a cross‐sectional, analytical, and descriptive pilot study that had as its target population family members of people who tested positive for HTLV‐1, treated from October 2017 to December 2019 at a health education unit for the diagnosis and multidisciplinary monitoring of PLHTLV in Belem, state of Para, Amazon region of Brazil. The inclusion criteria were families that had at least two people positive for HTLV‐1 infection, regardless of age group or degree of kinship.

The exclusion criteria were individuals who did not agree to participate and/or refused to sign the free and informed consent form (FICF). The variables investigated were age, sex, marital status, blood transfusion, breastfeeding, and condom use during sexual intercourse. Vertical transmission was suggested by a history of people who were breastfed and sexual transmission through a stable relationship lasting more than 3 months.

### 2.2. Detection of the HTLV‐1 Infection

From a blood sample, DNA extraction was performed from peripheral blood mononuclear cells (PBMCs) using the commercial kit (Wizard Genomic DNA Purification, Promega) according to the manufacturer’s instructions. All genomic DNA was subjected to amplification of the *human β-globin* gene with primers G73 (5′‐GAA​GAG​CCA​AAG​GAC​AGG​TAC‐3′) and G74 (5′‐CAA​CTT​CAT​CCC​TCA​CC‐3′), generating a 268‐bp fragment, where the objective was to assess DNA integrity and exclude the presence of PCR inhibitors [33]. In all stages of PCR in this study, positive and negative controls were used as reaction controls.

Partial amplification of the *pX* gene by nested PCR for detection of the proviral genome occurred according to a protocol adapted from Tuke et al. [34]. In the first nested PCR reaction, a solution containing 3.5 μL of Go Taq Green Master Mix (Promega, Madison, WI, USA), 1.0 μL of water, 0.25 μL (10 pmol) of each primer HTLV_external F (5′‐TTC​CCA​GGG​TTT​GGA​CGA​AG‐3′) (7219–7238, forward) and HTLV_external R (5′‐GGGTAAG GACCTTGAGGGTC‐3′) (7483–7464, reverse), and 2.0 μL of DNA for a final volume of 7 μL was made. It was subjected to a thermocycler for 35 cycles of 94°C, 52°C, and 72°C, generating a 259‐bp fragment [34]. In the second nested PCR reaction, 6.0 μL of Go Taq Green Master Mix (Promega, Madison, WI, USA), 0.25 μL (10 pmol) of each oligonucleotide, HTLV_internal F (5′‐CGG​ATA​CCC​AGT​CTA​CGT​GTT‐3′) (7248–7268, forward) and HTLV_internal R (5‐GAG​CCG​ATA​ACG​CGT​CCA​TCG‐3′) (7406–7386, reverse), 5.2 μL of water, and 0.3 μL of the byproduct of the first PCR were used, totaling a final volume of 12 μL that was subjected to a thermocycler for the same cycles as the first PCR step, generating a 159‐bp fragment [34].

Samples positive for the viral genome were subjected to the restrict fragment length polymorphism (RFLP) reaction to distinguish HTLV‐1 from other types of HTLV. The RFLP reaction of the *pX* gene product was performed from 6.0 μL of the amplified product, to which 2.8 μL of water, 1.1 μL of buffer E (Promega, Madison, WI, USA), and 0.1 μL of the restriction enzyme TaqI (10 U/μL, Promega, Madison, WI, USA) were added to a final volume of 10 μL that was incubated at 65°C for 2 h. The presence of the TaqI enzyme restriction site (T/CGA) generates 2 fragments (138 and 21 bp) in HTLV‐1 and 3 fragments (85, 53, and 21 bp) in HTLV‐2. The enzymatic digestion products were visualized through electrophoresis in a 3% agarose gel stained with ethidium bromide (0.5 mg/mL) using a transilluminator with an ultraviolet light source [35].

### 2.3. PCR for Sequencing

After HTLV‐1 infection confirmation, the nested PCR protocol of the 5′LTR region was performed to be used in nucleotide sequencing according to the adapted Seiki et al.’s (1983) protocol. In the first nested PCR reaction, a solution containing 8.3 μL of Go Taq Green Master Mix (Promega, Madison, WI, USA), 4.0 μL of water, 0.50 μL (10 pmol) of each primer LTR‐I.01 F (5′‐TGA​CAA​TGA​CCA​TGA​GCC​CCA​A‐3′) (1–22, forward) and LTR‐I.02 R (5′‐CGC​GGA​ATA​GGG​CTA​GCG​CT‐3′) (823–842, reverse), and 2.0 μL of DNA was made. It was subjected to a thermocycler for 25 cycles of 94°C, 62°C and 72°C, generating an 844‐bp fragment [36]. In the second nested PCR reaction, 13.6 μL of Go Taq Green Master Mix (Promega, Madison, WI, USA), 0.9 μL (20 pmol) of each primer LTR‐I.03 F (5′‐GGC​TTA​GAG​CCT​CCC​AGT​GA‐3′) (30–49, forward) and LTR‐I.04 R (5′‐GCC​TAG​GGA​ATA​AAG​GGG​CG‐3′) (781–800, reverse), 11.2 μL of water, and 0.5 μL of the byproduct of the first nested PCR were used. It was subjected to a thermocycler for 35 cycles of 94°C, 60°C, and 72°C [36].

### 2.4. Nucleotide Sequencing

The provirus genome was amplified using primers HTLV‐1 FG_O2S (5′‐CGC CGG GGC CTA CTT CCT AAC CAC ATC TGG CAA GG‐3′) and HTLV‐1 FG_O2R (5′‐TCT CCT GAG AGT GCT ATA GGA TGG GCT GTC GCT GGC TCC TAT‐3′), producing a 744‐bp fragment. This amplification was performed using GoTaq G2 Master Mix (Promega, Madison, WI, USA) with an annealing temperature of 55°C and 25 PCR cycles. The PCR products were then subjected to purification using the Wizard SV Gel and PCR Clean‐Up kit (Promega, Madison, WI, USA) as described by the manufacturer. Subsequently, the purified products were subjected to a sequencing reaction using the primers described above and the Big Dye Terminator Cycle Sequencing V.3.1 kit (Applied Biosystems, Foster City, CA, USA), following the manufacturer’s instructions. After this step, the products were purified using the BigDye XTerminator Purification kit (Applied Biosystems, Foster City, CA, USA) according to the manufacturer’s instructions. Finally, the final products were sequenced on the ABIPrism 3130xl platform (Applied Biosystems, Thermo Fisher, USA).

### 2.5. Nucleotide Sequences Analysis

All nucleotide sequences were compared with those deposited in the GenBank using the Basic Local Alignment Search Tool platform. Nucleotide sequences were analyzed using Unipro UGENE v 52.1 and MAFFT V.7. The analysis of the genetic diversity rate between the sequences of this study and those available in GenBank was performed using the BEAST V. 10.5.0 software, in a total of 25 samples containing all HTLV‐1 subtypes, generating comparative trees between them. This analysis was performed based on the Bayes theorem, which establishes the probability relationship between distinct events. The phylogenetic trees and the distance matrix were constructed using the IqTREE 2 program, and for the bootstrap analysis, 1000 replicates were considered to generate greater reliability for the grouping values, with the process being repeated 10 times. The trees were edited using the FigTree V.1.4.1 software. The families were numbered with their own numerical codes, with each family member receiving a letter according to the order in which they entered the clinic.

### 2.6. Ethics Statement

The investigations were conducted in accordance with the principles of Point 23 outlined in the Declaration of Helsinki (1975, revised in 2013) and in compliance with Resolution 466/2012 of the National Health Council of Brazil [37]. This study is part of the project “Researching infections and infectious diseases in university extension.” All methods were performed in accordance with the relevant guidelines and regulations. Our research was authorized by the Research Ethics Committee of the Center for Tropical Medicine of the Federal University of Para (process number: 1.218.417). All participants in this study received specialized medical follow‐up, care, and treatment at the Center for Tropical Medicine at the Federal University of Para.

Participants were included after being informed about the respective risks and benefits and after signing the informed consent form. Participants who were under 18 years of age were only included in this study after signed authorization by their respective guardians.

### 2.7. Statistical Analysis

Statistical analysis was performed using the Statistical Package for the Social Sciences (SPSS) Version 21.0 (SPSS, Chicago, Illinois), where Student’s *t*‐test, Fisher’s exact test, and chi‐square test were used to assess the association degree between the prevalence of HTLV‐1 infection and the study variables. Fisher’s exact test was performed to assess the frequency of vertical transmission according to the child’s gender. The 95% confidence interval (CI) considered the significance level *p* ≤ 0.05 for all analyses.

## 3. Results

Fourteen families with HTLV‐1 intrafamilial transmission participated in this study, with a total of 72 investigated members (14 index cases and 58 relatives). The frequency of HTLV‐1 infection was 56.94% (41/72) among all those investigated and 47% (27/58) in relatives of PLHTLV.

The median age of the investigated family members was 35 years (interquartile range: 25.0–51.5 years; range: 3–86 years). Among all those who tested positive for HTLV‐1 infection, 60.98% (25/41) were women, 78.05% (32/41, *p* = 0.005) were over 25 years old, 70.73% (29/41) declared themselves married, 85.37% (35/41, *p* = 0.033) declared no history of blood transfusion, 73.17% (30/41) reported having been breastfed in childhood, and condom use was reported only by 21.95% (9/41) of the individuals (Table 1).

**Table 1:** Epidemiological data from the 72 members of families with HTLV‐1 intrafamilial transmission in an endemic region of the Brazilian Amazon (*n* = 72).

Variables		Uninfected		Infected		*p* value^†^
		*N* = 31	%	*N* = 41	%	
Gender^b^	Female	18	58.06	25	60.98	0.994
	Male	13	41.94	16	39.02	

Age (years)^b^	≤ 25	9	29.03	5	12.19	0.005^∗^
	> 25	8	25.8	32	78.05	
	Uninformed	14		4		

Conjugal status^a^	Married	5	16.13	29	70.73	0.120
	Single	3	9.68	4	9.76	
	Widowed	1	3.22	1	2.44	
	Uninformed	22	70.97	7	17.07	

Blood transfusion history^b^	Yes	0	0	6	14.63	0.033^∗^
	No	31	100	35	85.37	

Breastfeeding history^b^	Yes	12	38.71	30	73.17	0.224
	No	1	3.23	—	—	
	Not sure	18	58.06	11	26.83	

Condom use^a^	Yes	1	3.23	9	21.95	1.000
	No	4	12.9	24	58.54	
	Uninformed	26	83.87	8	19.51	

*Note:* Variables adjusted to each other in each group.

Abbreviation: 95% CI, 95% confidence interval.

^∗^Statistically significant *p* value.

^a^Current variables.

^b^Anamnesis variables.

^†^Fisher’s exact test.

Based on epidemiological data, from the 10 families investigated for the occurrence of vertical transmission, seven (70%) presented this possibility (FAM5, FAM11, FAM13, FAM119, FAM151, FAM156, and FAM168), in which a frequency of 39% (*n* = 14) of potential vertical transmissions was identified in the 36 mother/child relationships. From the 12 families (FAM5, FAM11, FAM13, FAM30, FAM43, FAM48, FAM110, FAM119, FAM156, FAM158, FAM162, and FAM167) investigated for sexual transmission of HTLV‐1, 11 (92%) presented this possibility (except FAM119). Among the 19 marital relationships investigated, probable sexual transmission was observed in 14 (74%). The frequency of HTLV‐1 infection transmission in marital relationships (74%) was significantly higher (*p* = 0.0299) than in mother/child relationships (39%).

**Figure 1:**
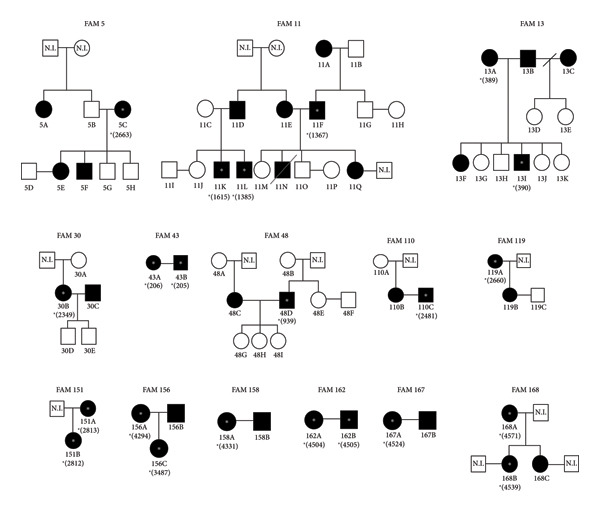
Pedigrees illustrating HTLV‐1 intrafamilial transmission routes across 14 families (FAM05 to FAM168) in an endemic region of the Brazilian Amazon.

Of the 41 individuals infected with HTLV‐1, 17 (42%) belonged to three families (FAM05, FAM11 and FAM 13), with an average of 12 individuals investigated and 5.7 individuals infected per family, while the remaining 11 families had an average of three individuals investigated and 2.2 infected per family. Only in these three families (FAM05, FAM11, and FAM 13) was it possible to identify both intrafamilial transmission routes, vertical and sexual (Figure 1).

The diagram visualizes the observed patterns of HTLV‐1 transmission, including sexual and vertical routes, and the familial aggregation of the HTLV‐1 Cosmopolitan subtype aA. Key to pedigree conventions: circles represent female individuals, and squares represent male individuals. Solid black symbols denote individuals with confirmed HTLV‐1 infection (seropositive and provirus detected), while open white symbols denote uninfected individuals. An asterisk (^∗^) indicates a sequenced sample, with the number in parentheses being the corresponding identifier in the laboratory database. N.I. denotes not investigated/no information. A single horizontal line (−) represents a conjugal relationship (potential sexual transmission route), and a vertical line (|) represents direct descent (potential vertical transmission route). Transmission route profile: mixed routes (both sexual and vertical transmission) were identified in families FAM05, FAM11, and FAM13. Predominantly sexual (horizontal) transmission was observed in families FAM30, FAM43, FAM48, FAM110, FAM119, FAM158, FAM162, and FAM167. Predominantly vertical transmission was observed in families FAM151, FAM156, and FAM168. Molecular analysis note: the 22 sequenced HTLV‐1 provirus samples (indicated by ^∗^) were obtained using the 5′LTR nested PCR protocol, according to Seiki et al. (1983), confirming the HTLV‐1 Cosmopolitan subtype aA and demonstrating low nucleotide diversity (0–3 differences).

This matrix presents the absolute number of nucleotide differences (p‐distance) between pairs of sequences obtained from infected members of the 14 families. The numerical codes in the first row and column correspond to the laboratory database identifiers for the sequenced samples. Genetic stability was observed in the HTLV‐1 sequences with an absence of nucleotide divergence (0%) in vertical transmissions (mother–child pairs) (Table 2).

**Table 2:** Nucleotide diversity matrix of HTLV‐1 sequences from individuals suggestive of intrafamilial transmission.

	1367 F.11	1385 F.11	1615 F.11	205 F.43	206 F.43	2812 F.151	2813 F.151	3487 F.156	4294 F.156	389 F.13	390 F.13	4504 F.162	4505 F.162	4539 F.168	4571 F.168
1367 F.11	0	2	2	3	3	2	2	3	2	2	2	3	3	2	2
1385 F.11	2	0	0	2	2	1	1	3	2	1	1	2	2	2	2
1615 F.11	2	0	0	2	2	1	1	3	2	1	1	2	2	2	2
205 F.43	3	2	2	0	1	2	2	3	2	2	2	3	3	3	3
206 F.43	3	2	2	1	0	1	1	3	2	1	1	2	3	2	2
2812 F.151	2	1	1	2	1	0	0	3	2	1	1	2	2	2	2
2813 F.151	2	1	1	2	1	0	0	3	2	1	1	2	2	2	2
3487 F.156	3	3	3	3	3	3	3	0	0	2	2	3	3	3	3
4294 F.156	2	2	2	2	2	2	2	0	0	1	1	2	2	2	2
389 F.13	2	1	1	2	1	1	1	2	1	0	0	2	2	1	1
390 F.13	2	1	1	2	1	1	1	2	1	0	0	2	2	1	1
4504 F.162	3	2	2	3	2	2	2	3	2	2	2	0	1	2	2
4505 F.162	3	2	2	3	3	2	2	3	2	2	2	1	0	3	3
4539 F.168	2	2	2	3	2	2	2	3	2	1	1	2	3	0	0
4571 F. 168	2	2	2	3	2	2	2	3	2	1	1	2	3	0	0

## 4. Discussion

In this pilot study, we found HTLV‐1 intrafamilial transmission in all 14 families treated at the health education unit in Belem, state of Para, Amazon region of Brazil. Infection was significantly higher in individuals over 25 years old and in those who reported no history of blood transfusion. The HTLV‐1 prevalence is higher in women than in men and increases with age [38]. The prevalence of HTLV‐1 infection in women was not significant in the present study, which may be related to a limited sample size.

The few reports that use this research model based on familial aggregation of HTLV‐1 infection have found a high frequency of transmission among family members of PLHTLV‐1 in the Amazon region [25] and in the State of Bahia [31, 39], as well as in international studies from Japan [40], Benin, West Africa [41], Taiwan [42], and Argentina [15].

Our results identified that 92% of families presented sexual transmission of HTLV‐1 infection. Furthermore, the frequency of HTLV‐1 infection transmission in marital relationships was significantly higher than in mother‐to‐child relationships. This significantly higher frequency of sexual transmission was also reported by another study from the Amazon region [25]. These findings highlight sexual transmission as the most evident route of intrafamilial viral dissemination in the region.

However, 70% of families with mother/child relationships had vertical transmission, confirming that this is the main route for perpetuating the virus in different generations of the same family. Data suggest that approximately 18%–26% of children from HTLV‐1‐positive mothers acquire the infection [14]. Continuous breastfeeding by a mother who is a carrier of the virus increases the chance of acquiring the infection in the infant by 14 times, especially when the period is longer than three continuous months and the mother is over 35 years old [43, 44].

The spread of the virus in Brazil was driven by the practice of breastfeeding, where, during the colonial period, it was common for enslaved African mothers to breastfeed not only their own children but also the children of white women for extended periods during the first years of life. In addition to the importance of vertical transmission in the initial viral spread, it is important to highlight the contribution of sexual transmission through the practice of unprotected sexual relations, whether marital or extramarital, to the intrafamilial transmission of HTLV. Later, in the 20th century, HTLV‐1 had a second period of intense introduction into Brazilian populations through Japanese migration, initially remaining in the southeast of Brazil, and then prevailing in the north of Brazil [45].

Brazil has a well‐designed health system based on a comprehensive, universal, participatory, and equitable health policy. Despite being an important achievement for the country’s democracy since the end of the 1980s, it was only in 2024 that measures to combat vertical transmission of HTLV were specifically targeted at pregnant women, parturients, and children exposed to the risk of vertical transmission. Until then, the epidemiology of HTLV‐1 remained unknown in Brazil due to the infection’s long latency periods and the absence of comprehensive public policies for screening, diagnosis, and treatment of related diseases [17]. Despite the high prevalence of this infection, ATL and HAM diagnoses remain low when compared to other endemic countries that have similar prevalence rates, such as Japan [46–48] and other countries in South America and the Caribbean [49].

Nucleotide divergence between HTLV‐1 sequences in cases of sexual transmission may occur due to the nature of the virus itself and the host’s immune response. The host’s immune system may exert selective pressure on the virus. During sexual transmission, the virus needs to adapt to a new immune environment in the new host, which may lead to the selection of variants that have advantageous mutations to escape the immune response [50].

All epidemiological studies of intrafamilial HTLV transmission, including this one, report significantly higher infection rates than those reported in population prevalence studies or investigations of specific groups. The two routes of intrafamilial transmission (vertical and sexual), as well as the highest average number of infected individuals, were identified only in the three families with the highest average number of investigated members. This means that the greater the number of family members investigated, the more evident the importance of intrafamilial aggregation in the spread of HTLV infection becomes. Notably, the stability observed in HTLV‐1 sequences in vertically transmitted infections and the presence of genetic diversity among sequences from sexual transmissions raise questions about the role of host genetic characteristics in viral transmission routes.

The genetic stability observed in the HTLV‐1 sequences is a crucial finding for understanding its pathobiology. The absence of nucleotide divergence (0%) in vertical transmissions (mother‐to‐child pairs), as clearly evidenced in Table 2, suggests a highly efficient and direct transmission process, most likely via infected T‐cells in breast milk or in utero. This cell‐to‐cell transfer mechanism shields the virus from the selective pressures of the systemic immune response during passage and establishment in the new host, acting as a “soft bottleneck” that permits the perpetuation of an identical genotype. In contrast, the slight divergence (0%–1%) observed in sexual transmissions can be attributed to the initial immune pressure within the recipient host. Viral exposure at mucosal surfaces, followed by replication and dissemination, may impose a more restrictive genetic bottleneck, leading to the selection of adaptive variants that, though minimally different, successfully establish infection and evade immune surveillance, reinforcing the underlying virological distinctions between the two routes.

The identification of the Cosmopolitan transcontinental subtype aA in all sequenced samples aligns with existing molecular and epidemiological surveillance data across South America. However, the high rates of intrafamilial transmission (47% in relatives) and the prominence of both sexual (74%) and vertical (39%) routes found in this pilot study focusing on family clusters underscore the urgent need for family‐centric public health policies in the Amazon. The endemic nature of HTLV‐1, coupled with the genetic stability of the subtype, suggests that prevention interventions must be multifaceted, encompassing not only prenatal screening to halt vertical transmission but also couples counseling (to prevent sexual transmission) and ring‐screening of close relatives. The results compellingly demonstrate that HTLV‐1 infection in the Para region is primarily perpetuated through the family unit, thus warranting larger, population‐based epidemiological studies to support effective strategies for breaking the transmission cycle.

## 5. Conclusion

We conclude that HTLV‐1 infection in Belem, Para region, is maintained by intense familial aggregation. Sexual transmission is confirmed as the primary driver for horizontal viral dissemination, while vertical transmission is the fundamental mechanism for perpetuating the endemicity across family generations. Molecular data strongly indicate the notable genetic stability of the Cosmopolitan transcontinental subtype aA. This is evidenced by little nucleotide variability among sequenced samples, with zero divergence observed in vertical transmission, suggesting minimal selective pressure on viral variants transferred to offspring. These epidemiological and molecular results emphasize that the family unit serves as the main reservoir for transmission in the Amazon. Therefore, it is imperative that future public health policies integrate prenatal screening with targeted ring‐screening of sexual partners and close relatives of infected individuals. This is crucial for eliminating intrafamilial transmission and effectively reducing the burden of HTLV‐1‐associated diseases [51].

## Ethics Statement

Our research was authorized by the Research Ethics Committee of the Center for Tropical Medicine of the Federal University of Pará (Process number: 1,218,417).

## Consent

The consent of the study participants was collected after reading, interpreting, and signing the consentiment term.

## Disclosure

The manuscript was submitted as a preprint (https://www.authorea.com/users/900825/articles/1276179-stability-and-perpetuation-of-human-t-lymphotropic-virus-1-htlv-1-in-intrafamilial-transmissions-of-infection-in-an-endemic-region-of-the-brazilian-ama2.zon).

## Conflicts of Interest

The authors declare no conflicts of interest.

## Author Contributions

Cássia Cristine Costa Pereira: research and writing–review and editing (equal). Milena Cristina Martins da Silva: translation (equal). Samir Mansour Moraes Casseb, Louise de Souza Canto Covre, Edna Aoba Yassui Ishikawa, and Maisa Silva de Sousa: methodology (equal). Carlos Araújo da Costa: formal analysis and research (lead). Maria de Nazaré Lima dos Reis: conceptualization (equal). Maisa Silva de Sousa and Leonardo Miranda dos Santos: conceptualization (lead), formal analysis, research (lead), writing–original draft preparation, and writing–review and editing (equal).

## Funding

This research was supported by the UFPA Pro‐Rectory for Extension and the Brazilian National Council for Research and Development — CNPq (MCTI/CNPQ/Universal 14/2014 _PROJ_459352/2014–8).

## Data Availability

The data that support the findings of this study are available on request from the corresponding author. The data are not publicly available due to privacy or ethical restrictions.

## References

[1] Poiesz B. J. , Ruscetti F. W. , Gazdar A. F. , Bunn P. A. , Minna J. D. , and Gallo R. C. , Detection and Isolation of Type C Retrovirus Particles From Fresh and Cultured Lymphocytes of a Patient With Cutaneous T-Cell Lymphoma, Proceedings of the National Academy of Sciences of the United States of America. (1980) 77, no. 12, 7415–7419, 10.1073/pnas.77.12.7415, 2-s2.0-0019254359. 6261256 PMC350514

[2] Kalyanaraman V. S. , Sarngadharan M. G. , Robert-Guroff M. , Miyoshi I. , Golde D. , and Gallo R. C. , A New Subtype of Human T-Cell Leukemia Virus (HTLV-II) Associated With a T-Cell Variant of Hairy Cell Leukemia, Science.(1982) 218, no. 4572, 571–573, 10.1126/science.6981847, 2-s2.0-0019907946. 6981847

[3] Forlani G. , Shallak M. , Accolla R. S. , and Romanelli M. G. , HTLV-1 Infection and Pathogenesis: New Insights From Cellular and Animal Models, International Journal of Molecular Sciences. (2021) 22, no. 15, 10.3390/ijms22158001. PMC8347336 34360767

[4] Bangham C. R. , Araujo A. , Yamano Y. , and Taylor G. P. , HTLV-1-associated myelopathy/tropical Spastic Paraparesis, Nature Reviews Disease Primers. (2015) 1, no. 1, 10.1038/nrdp.2015.12, 2-s2.0-85017226260. 27188208

[5] Tsukasaki K. and Tobinai K. , Biology and Treatment of HTLV-1 Associated T-Cell Lymphomas. Best Practice and Research, Clinical Haematology. (2013) 10.1016/j.beha.04.001. 23768636

[6] Bravo F. G. , Infective Dermatitis: A Purely Cutaneous Manifestation of HTLV-1 Infection, Seminars in Diagnostic Pathology. (2020) 10.1053/j.semdp.2019.04.002, 2-s2.0-85064389614. 31010607

[7] Rosadas C. and Taylor G. P. , Mother-To-Child HTLV-1 Transmission: Unmet Research Needs, Frontiers in Microbiology. (2019) 10.3389/fmicb.2019.00999, 2-s2.0-85068759417. PMC6517543 31134031

[8] Moriuchi M. and Moriuchi H. , Seminal Fluid Enhances Replication of Human T-Cell Leukemia Virus Type 1: Implications for Sexual Transmission, Journal of Virology. (2004) 78, 12709–12711, 10.1128/jvi.78.22.12709-12711. 15507662 PMC525095

[9] Tezuka K. , Fuchi N. , Okuma K. et al., HTLV-1 Targets Human Placental Trophoblasts in Seropositive Pregnant Women, Journal of Clinical Investigation. (2020) 130, 6171–6186, 10.1172/JCI135525. 33074247 PMC7598071

[10] Piron M. , Salvador F. , Caballero E. et al., HTLV-1/2 Infection in Blood Donors From a Non-Endemic Area (Catalonia, Spain) Between 2008 and 2017: A 10-Year Experience, Viruses. (2020) 14, 10.3390/v14091975. PMC9504911 36146780

[11] Miranda C. , Utsch-Gonçalves D. , Piassi F. C. C. et al., Prevalence and Risk Factors for Human T-Cell Lymphotropic Virus (HTLV) in Blood Donors in Brazil—A 10-Year Study (2007–2016), Frontiers of Medicine. (2022) 9, 10.3389/fmed.2022.844265. PMC8959844 35355612

[12] Futsch N. , Mahieux R. , and Dutartre H. , HTLV-1, the Other Pathogenic yet Neglected Human Retrovirus: From Transmission to Therapeutic Treatment, Viruses. (2018) 10.3390/v10010001, 2-s2.0-85038893569. PMC5795414 29267225

[13] Barr R. S. , Drysdale S. B. , Boullier M. et al., A Review of the Prevention of Mother-to-Child Transmission of Human T-Cell Lymphotrophic Virus Type 1 (HTLV-1) with a Proposed Management Algorithm, Frontiers of Medicine. (2022) 10.3389/fmed.2022.941647. PMC9304803 35872787

[14] Hino S. , Establishment of the milk-borne Transmission as a Key Factor for the Peculiar Endemicity of Human T-lymphotropic Virus Type 1 (HTLV-1): The ATL Prevention Program Nagasaki, Proceedings of the Japan Academy Series B: Physical and Biological Sciences. (2011) 10.2183/pjab.87.152, 2-s2.0-80052461308. PMC3149377 21558754

[15] Frutos M. C. , Gastaldello R. , Balangero M. et al., Silent Dissemination of HTLV-1 in an Endemic Area of Argentina. Epidemiological and Molecular Evidence of Intrafamilial Transmission, PLoS One. (2017) 12, 10.1371/journal.pone.0174920, 2-s2.0-85017163557. PMC5383099 28384180

[16] Brasil. Prevalência Da Infecção Por HTLV-1/2 No Brasil. Boletim Epidemiológico. Departamento De HIV/Aids, Tuberculose, Hepatites Virais e Infecções Sexualmente Transmissíveis, 2022, https://www.gov.br/saude/pt-br/media/pdf/2020/dezembro/11/boletim_epidemiologico_svs_48.pdf.

[17] Brasil, Portaria GM/MS No 3.148, De 6 De Fevereiro De 2024, Ministério da Saúde. (2024) https://goias.gov.br/saude/wp-content/uploads/sites/34/2024/02/PORTARIA-GM_MS-No-3.148.pdf%20Available.

[18] Rosadas C. , Menezes M. L. B. , Castro B. G. , Assone T. , Miranda A. E. et al., Blocking HTLV-1/2 Silent Transmission in Brazil: Current Public Health Policies and Proposal for Additional Strategies, PLoS Neglected Tropical Diseases. (2021) 10.1371/journal.pntd.0009717. PMC8460035 34555019

[19] Miranda A. E. , Rosadas C. , Assone T. , Pereira G. F. M. , Vallinoto A. C. R. , and Ishak R. , Strengths, Weaknesses, Opportunities and Threats (SWOT) Analysis of the Implementation of Public Health Policies on HTLV-1 in Brazil, Frontiers of Medicine. (2022) 9, 10.3389/fmed.2022.859115. PMC9021745 35462992

[20] Lopes F. T. , Botelho B. J. S. , and Vallinoto A. C. R. , Prevalência Da Infecção Pelo HTLV-1/2 Em Três Municípios Da Região Metropolitana De Belém-Pa, Brazilian Journal of Infectious Diseases. (2022) 26, 10.1016/j.bjid.2021.102274.

[21] Ferreira L. S. C. , CostaI J. H. G. , CostaI C. A. et al., Human T-lymphotropic Virus Seroprevalence in Riparian Communities in the Northeastern Region of Pará State, Brazil, Rev Pan-Amaz Saude. (2010) 1, no. 3, 10.5123/S2176-62232010000300014.

[22] Lima A. C. R. D. , Lopes F. T. , Freitas V. D. O. et al., Prevalence and Risk Factors for HTLV-1/2 Infection Inriverside and Rural Populations of the State of Pará, Viruses. (2022) 14. 10.3390/v14102262 PMC9610156 36298817

[23] Alencar S. P. , Souza M. C. , Fonseca R. R. S. et al., Prevalence and Molecular Epidemiology of Human T-Lymphotropic Virus (HTLV) Infection in People Living with HIV/AIDS in the Pará State, Amazon Region of Brazil, Frontiers in Microbiology. (2020) 11, 10.3389/fmicb.2020.572381. PMC7642294 33193170

[24] Ishak R. , Ishak M. O. G. , Azevedo V. N. et al., Detection of HTLV-IIa in Blood Donors in an Urban Area of the Amazon Region of Brazil (Belém, PA), Revista da Sociedade Brasileira de Medicina Tropical [em linha]. (1998) 31, no. 2, 193–197, 10.1590/s0037-86821998000200005. 9608238

[25] Costa C. A. D. , Furtado K. C. Y. O. , Ferreira L. S. C. et al., Familial Transmission of Human T-Cell Lymphotrophic Virus: Silent Dissemination of an Emerging but Neglected Infection, PLoS Neglected Tropical Diseases. (2013) 7, 10.1371/journal.pntd.0002272, 2-s2.0-84879528131. PMC3681619 23785534

[26] Silva I. C. , Pinheiro B. T. , Nobre A. F. S. et al., Moderada Endemicidade Da Infecção Pelo Vírus linfotrópico-T Humano Na Região Metropolitana De Belém, Pará, Brasil, Revista Brasileira de Epidemiologia. (2018) 21, 10.1590/1980-549720180018, 2-s2.0-85055076578. 30328937

[27] Nobre A. , Almeida D. S. , Ferreira L. et al., Low Genetic Diversity of the Human T-Cell Lymphotropic Virus (HTLV-1) in an Endemic Area of the Brazilian Amazon Basin, PLoS One. (2018) 13. 10.1371/journal.pone.0194184 PMC5860735 29558516

[28] Brito W. R. , Cardoso-Costa G. L. , Junior L. M. R. et al., Prevalence and Risk Factors for HTLV-1/2 Infection in Quilombo Remnant Communities Living in the Brazilian Amazon, Frontiers in Public Health. (2022) 10. 10.3389/fpubh.2022.871865 PMC9005874 35433598

[29] Paiva A. , Smid J. , Haziot M. E. J. , Assone T. A. , Pinheiro S. et al., High Risk of Heterosexual Transmission of Human T-Cell Lymphotropic Virus Type 1 Infection in Brazil, Journal of Medical Virology. (2017) 89, 1287–1294, 10.1002/jmv.24745, 2-s2.0-85007292787. 27935065

[30] Oliveira-Filho A. B. , Frade P. C. R. , Fonseca R. R. S. et al., Spread of Human T-Lymphotropic Virus 1 and 2 Among Relatives of People Who Use Illicit Drugs in Northern Brazil, Frontiers in Microbiology. (2022) 13, 10.3389/fmicb.2022.889948. PMC9205188 35722295

[31] Silva A. N. D. , Araújo T. , Boa-Sorte N. et al., Epidemiological and Molecular Evidence of Intrafamilial Transmission Through Sexual and Vertical Routes in Bahia, the State With the Highest Prevalence of HTLV-1 in Brazil, PLoS Neglected Tropical Diseases. (2023) 17. 10.1371/journal.pntd.0011005 PMC10593241 37769013

[32] Bandeira L. M. , Puga M. A. , Weis-Torres S. M. S. et al., Human T-Cell Leukemia Virus Type 1 Infection Among Japanese Immigrants and Their Descendants Living in Southeast Brazil: A Call for Preventive and Control Responses, PLoS Neglected Tropical Diseases. (2021) 15, 10.1371/journal.pntd.0009066. PMC7864455 33544713

[33] Greer C. E. , Peterson S. L. , and Kiviat N. B. , PCR Amplification From Paraffin-Embedded Tissues. Effects of Fixative and Fixation Time, American Journal of Clinical Pathology. (1991) 95, 117–124, 10.1093/ajcp/95.2.117, 2-s2.0-0026099836. 1846996

[34] Tuke P. W. , Luton P. , and Garson J. A. , Differential Diagnosis of HTLV-I and HTLV-II Infections by Restriction Enzyme Analysis of “Nested” PCR Products, Journal of Virological Methods. (1992) 40, 163–173, 10.1016/0166-0934(92)90065-L, 2-s2.0-0026454755. 1452632

[35] Segurado A. A. , Biasutti C. , Zeigler R. et al., Identification of Human T-lymphotropic Virus Type I (HTLV-I) Subtypes Using Restrited Fragment Length Polymorphism in a Cohort of Asymptomatic Carriers and Patients With HTLV-I-associated myelopathy/tropical Spastic Paraparesis From São Paulo, Brazil, Memorias Do Instituto Oswaldo Cruz. (2002) 97, 329–333, 10.1590/S0074-02762002000300009, 2-s2.0-0036547929. 12048560

[36] Seiki M. , Hattori S. , and Yoshida M. , Human Adult T-Cell Leukemia Virus: Complete Nucleotide Sequence of the Provirus Genome Integrated in Leukemia Cell DNA, Proceedings of the National Academy of Sciences of the United States of America. (1983) 80, 3618–3622, 10.1073/pnas.80.12.3618, 2-s2.0-1542686422. 6304725 PMC394101

[37] Brasil. Conselho Nacional de Saúde (Brasil) , Resolução N° 466, De 12 De Dezembro De 2012, 2012, Brasília, http://www.conselho.saude.gov.br/web_comissoes/conep/index.html.

[38] Maloney E. M. , Murphy E. L. , Figueroa P. et al., Human T-lymphotropic Virus Type I (HTLV-I) Seroprevalence in Jamaica, American Journal of Epidemiology. (1991) 133, no. 11, 1125–1134, 10.1093/oxfordjournals.aje.a115825, 2-s2.0-0025825897. 2035516

[39] Mello M. A. , Conceição A. F. , Sousa S. M. B. et al., HTLV-1 in Pregnant Women From the Southern Bahia, Brazil: A Neglected Condition Despite the High Prevalence, Virology Journal. (2014) . 10.1186/1743-422X-11-28 PMC3974122 24524416

[40] Itabashi K. , Miyazawa T. , Sekizawa A. et al., A Nationwide Antenatal Human T-Cell Leukemia Virus Type-1 Antibody Screening in Japan, Frontiers in Microbiology. (2020) 11, 10.3389/fmicb.2020.00595.eCollection. PMC7160230 32328047

[41] Houinato D. , Preux P. M. , Charriere B. et al., Interest of LQAS Method in a Survey of HTLV-I Infection in Benin (West Africa), Journal of Clinical Epidemiology. (2002) 55, no. 2, 192–196, 10.1016/s0895-4356(01)00463-2, 2-s2.0-0036140902. 11809358

[42] Lu S. C. , Kao C. L. , Chin L. T. et al., Intrafamilial Transmission and Risk Assessment of HTLV-I Among Blood Donors in Southern Taiwan, The Kaohsiung Journal of Medical Sciences. (2001) 17, no. 3, 126–132. 11486644

[43] Boostani R. , Sadeghi R. , Sabouri A. , and Ghabeli-Juibary A. , Human T-lymphotropic Virus Type I and Breastfeeding; Systematic Review and meta-analysis of the Literature, Current Journal of Neurology. (2019) 10.18502/ijnl.v17i4.589. PMC6555888 31210902

[44] Rousseau C. M. , Nduati R. W. , Richardson B. A. et al., Longitudinal Analysis of Human Immunodeficiency Virus Type 1 RNA in Breast Milk and of Its Relationship to Infant Infection and Maternal Disease, Journal of Infectious Diseases. (2003) 187, 741–747, 10.1086/374273, 2-s2.0-0037333250. 12599047 PMC3384731

[45] Comissão de Recenseamento da Colonia Japonesa , The Japanese Immigrant in Brazil, 1964, The University of Tokyo Press.

[46] Iwanaga M. , Epidemiology of HTLV-1 Infection and ATL in Japan: An Update, Frontiers in Microbiology. (2020) 10.3389/fmicb.2020.01124. PMC7273189 32547527

[47] Rosadas C. , Sohler M. P. , Oliveira A. C. P. , Casseb J. , Sousa M. , and Taylor G. P. , Adult T-Cell leukaemia/lymphoma in Brazil: A Rare Disease or Rarely Diagnosed?, British Journal of Haematology. (2020) 188, no. 4, e46–e49, 10.1111/bjh.16318. 31743423

[48] Ito S. , Iwanaga M. , Nosaka M. et al., Epidemiology of Adult T-Cell leukemia-lymphoma in Japan: An Updated Analysis, 2012-2013, Cancer Science. (2021) 112, no. 10, 4346–4354, 10.1111/cas.15097. 34355480 PMC8486190

[49] Oliveira P. D. , de Carvalho R. F. , and Bittencourt A. L. , Adult T-Cell leukemia/lymphoma in South and Central America and the Caribbean: Systematic Search and Review, International Journal of STD & AIDS. (2017) 10.1177/0956462416684461, 2-s2.0-85012206963. 28178905

[50] Abdelmoumen K. , Alsibai K. D. , Rabier S. et al., Adult T-Cell Leukemia and Lymphoma in French Guiana: A Retrospective Analysis With Real-Life Data From 2009 to 2019, Lancet Reg Health Am. Apr. (2023) 21, no. 21, 10.1016/j.lana.2023.100492. PMC10149364 37139265

[51] Rosadas C. , Malik B. , Taylor G. P. , and Puccioni-Sohler M. , Estimation of HTLV-1 Vertical Transmission Cases in Brazil per Annum, PLoS Neglected Tropical Diseases. (2018) 12, 10.1371/journal.pntd.0006913, 2-s2.0-85057534542. PMC6261628 30418973

